# Variability in Myosteatosis and Insulin Resistance Induced by High-Fat Diet in Mouse Skeletal Muscles

**DOI:** 10.1155/2014/569623

**Published:** 2014-08-14

**Authors:** Massimo Collino, Raffaella Mastrocola, Debora Nigro, Fausto Chiazza, Manuela Aragno, Giuseppe D'Antona, Marco A. Minetto

**Affiliations:** ^1^Department of Drug Science and Technology, University of Turin, Via Giuria 9, 10125 Turin, Italy; ^2^Department of Clinical and Biological Sciences, University of Turin, Corso Raffaello 30, 10125 Turin, Italy; ^3^Department of Molecular Medicine University of Pavia, Via Forlanini 6, 27100 Pavia, Italy; ^4^Division of Endocrinology, Diabetology and Metabolism, Department of Medical Sciences, University of Turin, Corso Dogliotti 14, 10126 Turin, Italy

## Abstract

Nutrient overload leads to impaired muscle oxidative capacity and insulin sensitivity. However, comparative analyses of the effects of dietary manipulation on skeletal muscles with different fiber composition are lacking. This study aimed to investigate the selective adaptations in the soleus and tibialis anterior muscles evoked by administration of high-fat diet for 12 weeks in 10 mice (HFD mice) compared to 10 animals fed with a normal chow diet (control mice). Mice fed with the HFD diet exhibited hyperlipidemia, hyperinsulinemia, hyperglycemia, and lower exercise capacity in comparison to control mice. In control mice, soleus fibers showed higher lipid content than tibialis anterior fibers. In contrast, the lipid content was similar between the two muscles in HFD mice. Significant differences in markers of muscle mitochondrial production and/or activity as well as of lipid synthesis were detected between HFD mice and control mice, especially in the tibialis anterior. Moreover, translocation of GLUT-4 transporter to the plasma membrane and activation of the insulin signaling pathway were markedly inhibited in the tibialis and slightly reduced in the soleus of HFD mice compared to control mice. Overall, these results show that adaptive responses to dietary manipulation occur in a muscle-specific pattern.

## 1. Introduction

Myosteatosis (also known as ectopic skeletal muscle adiposity) represents the fat infiltration within myocytes (intramyocellular fat) and within the fascia surrounding skeletal muscle (intermuscular fat) [1]. It has been observed that its association with muscle atrophy (also known as “fatty atrophy”) may result in impaired muscle oxidative capacity that, in turn, triggers a fast-to-slow transition of muscle fibers to enhance the muscle oxidative potential and/or mitochondrial content [2, 3]. Animal studies performed in diet-induced obesity models have suggested that a prolonged mismatch between intramyocellular lipid accumulation and adaptations in oxidative capacity of muscle fibers may ultimately result in impaired oxidation of lipids and glucose (i.e., mitochondrial dysfunction) that implies decreased ATP production and increased production of reactive oxygen species [2, 3], resulting in insulin resistance [4, 5]. Possible mechanisms underlying mitochondrial dysfunction are the increased saturation of phospholipids at the mitochondrial membranes, downregulation of genes involved in oxidative phosphorylation, and oxidative stress [2–5].

Given the well-known differences in oxidative capacity between different fiber types, it may be hypothesized that muscle-specific adaptations occur in response to high-fat-diet- (HFD-) induced myosteatosis. To our knowledge, so far, there is only one previous study investigating this hypothesis and showing that triglyceride accumulation, fast-to-slow transition, and impaired oxidative capacity in response to HFD occurred in a muscle-specific pattern in mice [3]. We hypothesized that the myosteatosis-induced changes in oxidative capacity of muscle fibers could also be associated with decreased exercise capacity and changes in muscle contractile properties and that these associations could occur in a muscle-specific manner predicting the development of muscle-specific insulin resistance. Therefore, the aim of this study was to investigate in two animal muscles (characterized by different fiber type composition such as tibialis anterior and soleus) the differences in HFD-induced myosteatosis and changes in muscle oxidative metabolism, contractile properties, and insulin sensitivity. We chose to study the diet-induced adaptations in two muscles characterized by high dependence on either oxidative metabolism, the soleus (which mostly contains type I fibers), or glycolytic metabolism, the tibialis anterior (which mostly contains fast type IIb and type IIa and a minority of type I fibers) [6–8].

## 2. Materials and Methods

### 2.1. Animals and Diet

Four-week-old male C57Bl6/J mice (Harlan-Italy, Udine, Italy) were housed in a controlled environment at 25 ± 2°C with alternating 12-h light and dark cycles. All the animals were fed with a normal pellet diet for 1 week prior to the experiment. The animals were then allocated to two dietary regimens: chow diet (control animals: *n* = 10) or high-fat diet (HFD animals: *n* = 10) for 12 weeks. The kinetics of dietary manipulation has been chosen according to previously published papers, showing that mice fed with a high-fat diet for 12 weeks were more susceptible to the development of insulin resistance, with a significant increase in intramuscular triglycerides [9, 10]. The HFD contained 45% fat, 20% protein, and 35% carbohydrate (Research Diets, New Brunswick, NJ). Body mass and intake of water and food were recorded weekly. Animal care was in compliance with Italian regulations on the protection of animals used for experimental and other scientific purposes (DM 116/92) and the experiment was approved by the Turin University Ethics Committee.

### 2.2. Oral Glucose Tolerance Test

One day before the mice were due to be killed, the oral glucose tolerance test (OGTT) was performed after a fasting period of 6 h by administering glucose (2 g/kg) by oral gavage. Once before administration and 15, 30, 60, and 120 min afterward, blood was obtained from the saphenous vein, and glucose concentration was measured with a conventional Glucometer (GlucoGmeter, Menarini Diagnostics, Florence, Italy).

### 2.3. Blood Biochemical Analysis

After 12 weeks of dietary manipulation, the mice were anaesthetised with i.p. injection (30 mg/kg) of Zoletil 100 (Laboratoires Virbac, France) and killed by aortic exsanguination. Blood samples were collected and plasma was isolated. Glycemia was measured using the GlucoGmeter kit and insulin levels were measured using an enzyme-linked immunosorbent assay (ELISA) kit (Mercodia AB, Uppsala, Sweden). The plasma lipid profile was determined by measuring triglycerides and total cholesterol by standard enzymatic procedures using reagent kits (Hospitex Diagnostics, Florence, Italy).

### 2.4. *In Vivo* Muscle Function Assessment

Exercise capacity was assessed using an exhaustion incremental treadmill test. This procedure was previously described in detail [11]. Briefly, each animal was placed on the belt of a 6-lane motorized treadmill (Exer 3/6 Treadmill, Columbus Instruments, OH, USA), supplied with shocker plates, which could be individually enabled or disabled for each lane. During each exhaustion test the electrical stimulus was fixed at 200 ms duration, 0.34 mA amplitude, and 1 Hz repetition rate.

After acclimatization, all mice were subjected to initial exhaustion treadmill tests at 0° inclination according to the following protocol: 5 min at 5 m/min and followed by incremental increase of speed of 1 m/min every min until exhaustion. Exhaustion was defined as spending time on the shocker plate without attempting to reengage the treadmill within 20 s. Three tests were performed on each animal, allowing 4 days between each test. Values obtained were averaged, providing a single value per animal. In a second set of experiments, endurance was evaluated as the time spent on the treadmill belt running at 50% of the maximal velocity reached in the previous exhaustion incremental test.

### 2.5. *Ex Vivo* Functional Assessment of Tibialis Anterior Muscle Preparations

The method used for mechanical analysis of intact muscles was described previously [12]. Briefly, the tibialis anterior muscle of the right leg was dissected, placed in an organ bath filled with Krebs solution (composition: NaCl 120 mM, KCl 2.4 mM, CaCl_2_ 2.5 mM, MgSO_4_ 1.2 mM, glucose 5.6 mM, KH_2_PO_4_ 1.2 mM, NaHCO_3_ 24.8 mM, and pH 7.4), bubbled with 95% O_2_ and 5% CO_2_ at a constant temperature of 22°C, and attached to a force transducer (Radnoti Organ Bath System, AD Instruments). Electrical pulses were delivered through platinum electrodes connected to a stimulator (Tumiati, Italy). Tetanic isometric contractions were evoked (110 Hz, 500 ms, supramaximal amplitude) at *L*_*o*_ (the length at which the maximal isometric force is observed) and twich time to peak and maximal tetanic force (Tf) were measured. Specific tetanic force (i.e., maximal tetanic force normalized for muscle volume: g/*μ*L) was considered for the comparison between control mice and HFD mice. Further, the fatigue index was measured (and expressed in %) as the tetanic force drop for different stimulation frequencies (0.03, 0.09, and 0.3 Hz) compared to the maximal tetanic force [8].

### 2.6. Muscle Extracts

The soleus and tibialis anterior muscles were isolated, weighed, and rapidly freeze-clamped with liquid nitrogen and stored at −80°C, as previously described [13]. Total extracts of soleus and tibialis anterior were obtained from 10% (w/v) homogenates in RIPA buffer containing 20 mmol/L TRIS-HCl pH 7.4, 150 mmol/L NaCl, 2 mmol/L EGTA, 1 mmol/L EDTA, 1% TRITON-X100, and protease inhibitors [1 mM dithiothreitol (DTT), 0.5 mM phenylmethyl sulphonyl fluoride (PMSF), 5 *μ*g/mL aprotinin, and 2.5 *μ*g/mL leupeptin]. After 40 minutes of incubation in ice, samples were sonicated and cleared by centrifugation at 15,000 g at 4°C for 40 min. Supernatants were removed and protein content was determined using the Bradford assay. Protein extracts were stored at −80°C until use.

### 2.7. Skeletal Muscle Lipid Content

Triglycerides were extracted from skeletal muscle homogenates and assayed using reagent kits according to the manufacturer's instructions (Triglyceride Quantification Kit, Abnova Corporation, Aachen, Germany). Intramyocellular lipid accumulation in the soleus and tibialis anterior was evaluated by Oil Red O staining on 10 *μ*m cryostatic sections. Stained tissues were viewed under an Olympus Bx4I microscope (40x magnification) with an AxioCamMR5 photographic attachment (Zeiss, Gottingen, Germany).

### 2.8. Western Blot Analysis

About 60 *μ*g of total proteins was loaded for Western blot experiments. Proteins were separated by 8% sodium dodecyl sulphate-polyacrylamide gel electrophoresis (SDS-PAGE) and transferred to a polyvinylidene difluoride (PVDF) membrane, which was then incubated with a primary antibody (rabbit anti-total GSK-3*β*, dilution 1 : 200; goat anti-pGSK-3*β* Ser^9^, dilution 1 : 200; rabbit anti-total Akt, dilution 1 : 1000; mouse anti-pAkt Ser^473^, dilution 1 : 1000; rabbit anti-total IRS-1, dilution 1 : 200; goat anti-pIRS-1 Ser^307^, dilution 1 : 200; rabbit anti-GLUT-4, dilution 1 : 2000; rabbit anti-total ACC, dilution 1 : 500; rabbit anti-pACC Ser^79^, dilution 1 : 1000; rabbit anti-CPT-11 m, dilution 1 : 200; and mouse anti-SDH-A, dilution 1 : 200). Blots were then incubated with a secondary antibody conjugated with horseradish peroxidase (dilution 1 : 10000) and developed using the ECL detection system. The immunoreactive bands were visualised by autoradiography and the density of the bands was evaluated densitometrically using Gel Pro Analyzer 4.5, 2000 software (Media Cybernetics, Silver Spring, MD, USA). The membranes were stripped and incubated with alpha-tubulin monoclonal antibody (dilution 1 : 5000) and subsequently with an anti-mouse antibody (dilution 1 : 10000) to assess gel-loading homogeneity.

### 2.9. Immunohistochemistry

For immunodetection of the glucose transporter type-4 (GLUT-4), immunohistochemical staining was performed on 10 *μ*m acetone fixed cryostatic sections of soleus and tibialis anterior muscles. Endogenous peroxidases were inactivated for 5 minutes with 3% H_2_O_2_. Sections were blocked for 30 minutes with 3% BSA in PBS. Thus, samples were incubated overnight with rabbit anti-GLUT-4 antibody and then for 1 hour with an anti-rabbit IgG-HRP conjugated secondary antibody. The specific staining was detected with diaminobenzidine and sections were visualized with Olympus-Bx4I microscope connected by a photographic attachment (Carl Zeiss, Oberkochen, Germany). A negative control was included in which the primary antibody was replaced with a nonimmune isotypic control antibody.

### 2.10. Materials

Unless otherwise stated, all compounds were purchased from the Sigma-Aldrich Company Ltd. (St. Louis, MI, USA). The BCA Protein Assay kit and SuperBlock blocking buffer were from Pierce Biotechnology Inc. (Rockford, IL, USA) and PVDF was from the Millipore Corporation (Bedford, MA, USA). Antibodies were from Cell-Signaling Technology (Beverly, MA, USA) and from Santa Cruz Biotechnology (Santa Cruz, CA, USA). Luminol ECL was from PerkinElmer (Waltham, MA, USA).

### 2.11. Statistical Analysis

The Mann-Whitney *U* test was used for comparisons between muscles (tibialis anterior versus soleus) and groups (control mice versus HFD mice). Data were expressed as mean ± standard deviation (SD). Threshold for statistical significance was set to *P* = 0.05. All statistical tests were performed with Statistica 6 (Statsoft Inc., Tulsa, OK, USA) software package.

## 3. Results

### 3.1. Effect of HFD on Body Mass, Muscle Mass, and Glycometabolic Balance

**Figure 1:**
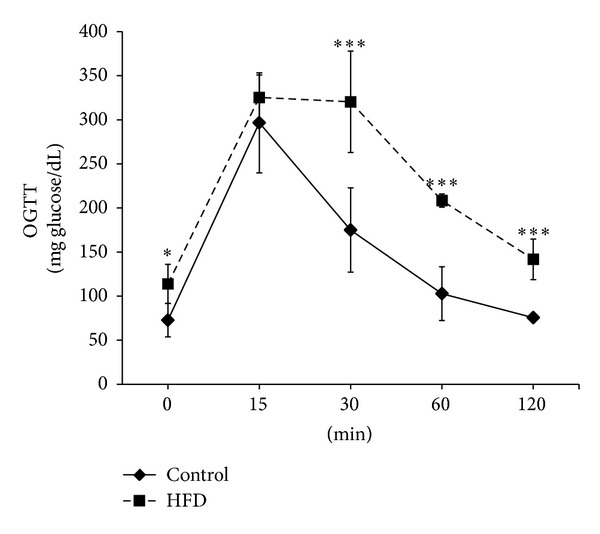
Effect of high-fat diet (HFD) on oral glucose tolerance in mice administered glucose (2 g*·*kg^−1^) by oral gavage. Values are means ± SD of 10 animals per group.

After 12 weeks of dietary manipulation, HFD mice had significantly higher body mass increase and lower tibialis and soleus mass in comparison to control mice (Table 1). Moreover, HFD caused significant increases in glucose, insulin, total cholesterol, and triglyceride levels. When compared to control animals, HFD mice showed more than 60% increase in fasting glucose levels, which was associated with almost 30% increase in insulin concentrations (Table 1). Consistently, HFD mice showed a significant impairment in glucose tolerance to exogenously administered glucose (Figure 1).

### 3.2. Effect of HFD on Muscle Function

**Figure 2:**
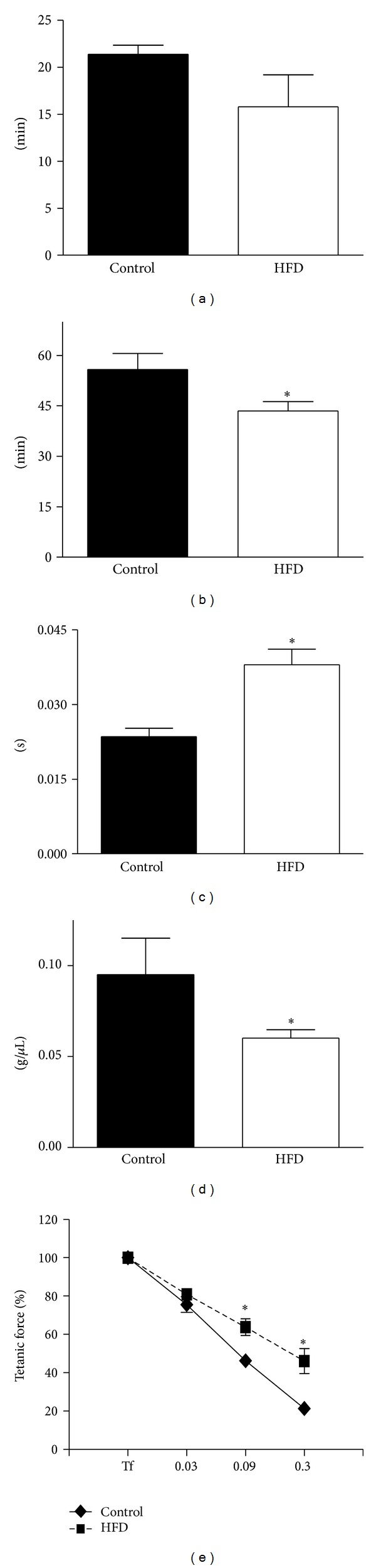
((a)-(b))* In vivo* muscle function determined by exhaustion incremental treadmill test and endurance treadmill test. Exhaustion time (a) and endurance time (b) are expressed in min. Control mice: closed bars; HFD mice: white bars. *P < 0.05 versus control mice, *n* = 5 experiments. All data are expressed as mean ± SD. ((c)–(e))* Ex vivo* functional parameters of tibialis intact preparations from control (closed bars or closed diamonds) and HFD mice (white bars or closed squares). (c) Twitch time to peak (s); (d) specific tetanic force (g/*μ*L); (e) fatigue index measured as the tetanic force drop for different stimulation frequencies (from 0.03 to 0.9 Hz) compared to the maximal tetanic force (Tf) and expressed in %. *P < 0.05 versus control mice, *n* = 5 experiments. All data are expressed as mean ± SD.

Muscle function was assessed* in vivo* by treadmill tests (exhaustion incremental treadmill test and endurance treadmill test): HFD mice showed reduced resistance to fatigue (Figures 2(a)-2(b)) compared to control mice.

To assess* ex vivo* muscle function, time to peak tension and specific tetanic force of the tibialis anterior muscle were considered: HFD mice showed a significantly higher time to peak tension (Figure 2(c)) and lower specific tetanic force (Figure 2(d)) in comparison to control animals. Moreover, the fatigue index was lower in HFD mice compared to control mice (Figure 2(e)).

### 3.3. Effect of HFD on Muscle Lipid Content

**Figure 3:**
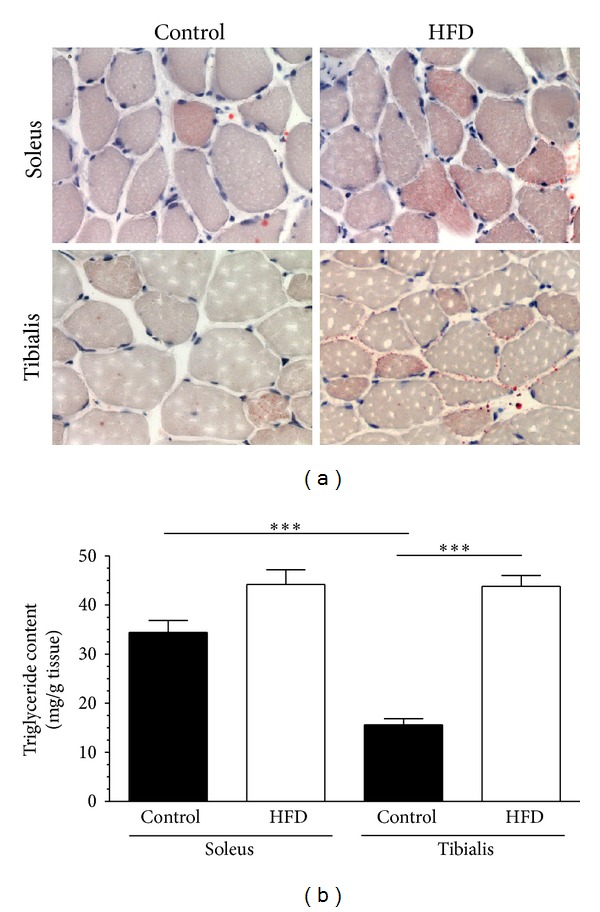
Effect of high-fat diet (HFD) on muscle histology and triglyceride content. Panel (a): representative photomicrographs (magnification 400x) of histological analyses performed in soleus and tibialis anterior sections of control mice and HFD mice with Oil Red O staining (performed on 10 animals per group). Panel (b): triglyceride content assessed in homogenates of soleus and tibialis anterior muscles of control mice and HFD mice. Values are means ± SD of 10 animals per group. ***P < 0.001.

Oil Red O staining sections highlighted low levels of lipid deposition in both soleus and tibialis anterior of control mice (Figure 3(a)). Moreover, soleus fibers contained higher levels of lipid droplets than tibialis anterior fibers in control mice. In contrast, the density of lipid droplets was similar between the two skeletal muscles of HFD mice, thus suggesting a higher increase in lipid content in tibialis anterior than in soleus after 12 weeks of dietary manipulation. As expected, the qualitative differences in the histological evaluation of muscle lipid deposition were confirmed by measurement of skeletal muscle triglyceride content (Figure 3(b)). In control mice, the triglyceride content was doubled in soleus when compared to anterior tibialis. Interestingly, HFD evoked a threefold increase in tibialis triglyceride accumulation and less than 30% increase in soleus triglyceride accumulation (in comparison to control mice), thus resulting in no significant differences in triglyceride content between the two muscles after dietary manipulation.

### 3.4. Effect of HFD on Muscle Oxidative Metabolism

**Figure 4:**
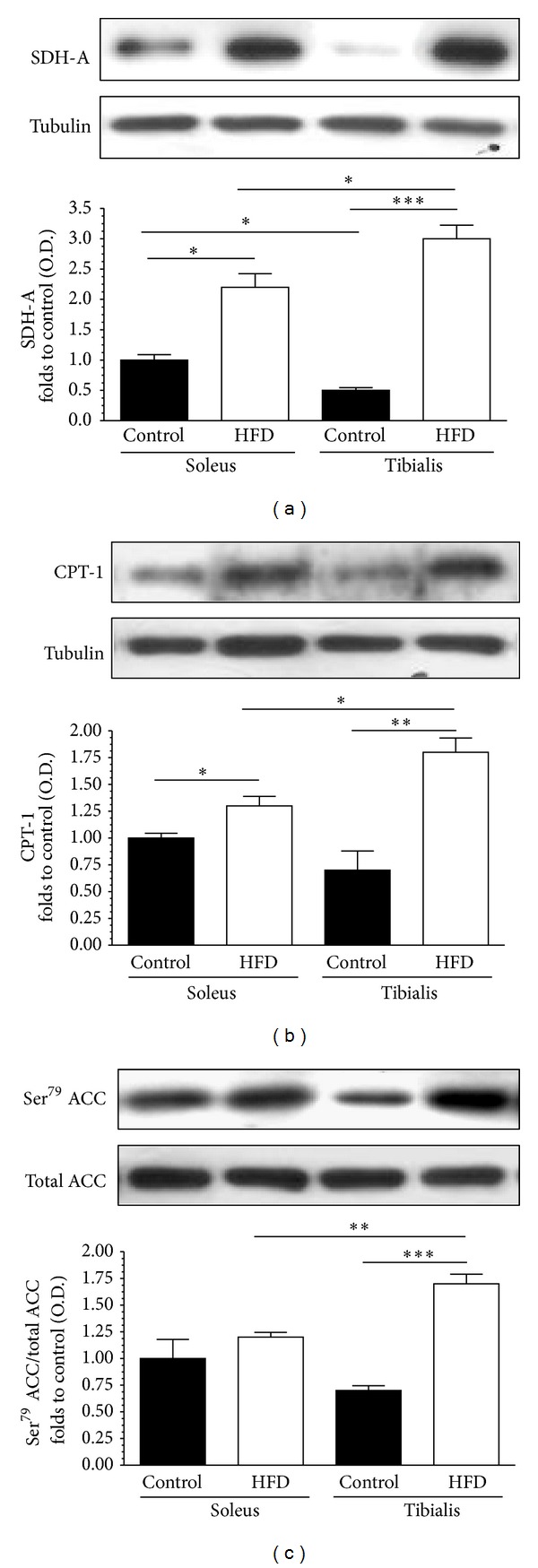
Effect of high-fat diet (HFD) on lipogenesis and lipid oxidation. Succinate dehydrogenase (SDH) activity (panel (a)), citrate transport protein (CTP)-1 expression (panel (b)), and total acetyl-CoA carboxylase (ACC) protein expression and Ser^79^ phosphorylation (panel (c)) were analyzed by Western blot on muscle homogenates. Densitometric analysis of the bands is expressed as relative optical density (O.D.), corrected for the corresponding tubulin contents and normalized using the related control band. The data are means ± SD of 10 animals. *P < 0.05; **P < 0.01; ***P < 0.001.

To elucidate the molecular mechanism(s) underlying the differences in skeletal muscle triglyceride accumulation, selective changes in markers of muscle mitochondrial production and/or activity were evaluated. Specifically, the expression of the succinate dehydrogenase complex subunit-A (SDH-A), a typical marker of mitochondrial density, and carnitine palmitoyl transferase-1 (CPT-1), a key regulatory enzyme in mitochondrial *β*-oxidation, as well as the phosphorylation/activation of acetyl-CoA carboxylase (ACC), whose central role in the regulation of lipid synthesis is well known, were measured on both soleus and tibialis homogenates by immunoblotting. As reported in Figure 4, HFD evoked a slight increase in protein expression of SDH-A and CPT-1 in soleus homogenates. Notably, a more relevant increase in the same markers was recorded in the tibialis anterior of HFD mice when compared to control mice. When the activation of the ACC pathway was assessed, induction of Ser^79^ phosphorylation of ACC was massive in tibialis anterior of HFD mice compared to control mice, whereas no significant differences between the two groups of animals were observed in soleus homogenates.

### 3.5. Effect of HFD on GLUT-4 Translocation and Insulin Signaling Pathway

**Figure 5:**
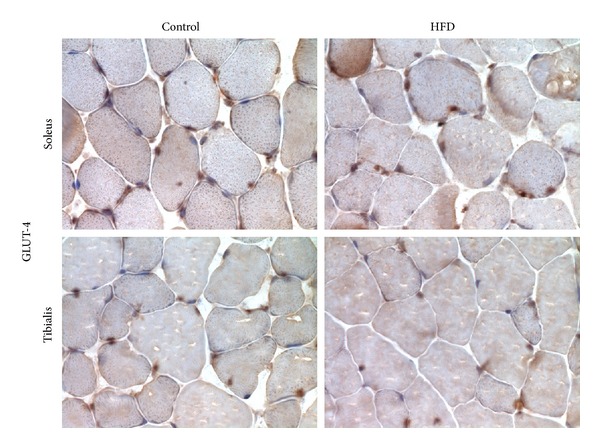
Representative photomicrographs (magnification 400x) showing the effect of high-fat diet (HFD) on GLUT-4 membrane translocation in the soleus and tibialis anterior muscles of control mice and HFD mice (assessed by immunohistochemistry on 10 animals per group).

As shown in Figure 5, GLUT-4 was detected in both soleus and tibialis anterior of control mice, with highest levels in soleus. A reduction in GLUT-4 immunostaining was observed in muscle sections of HFD mice. Specifically, translocation of GLUT-4 transporter to the plasma membrane was markedly inhibited in the tibialis and slightly reduced in the soleus of HFD mice compared to control animals.

**Figure 6:**
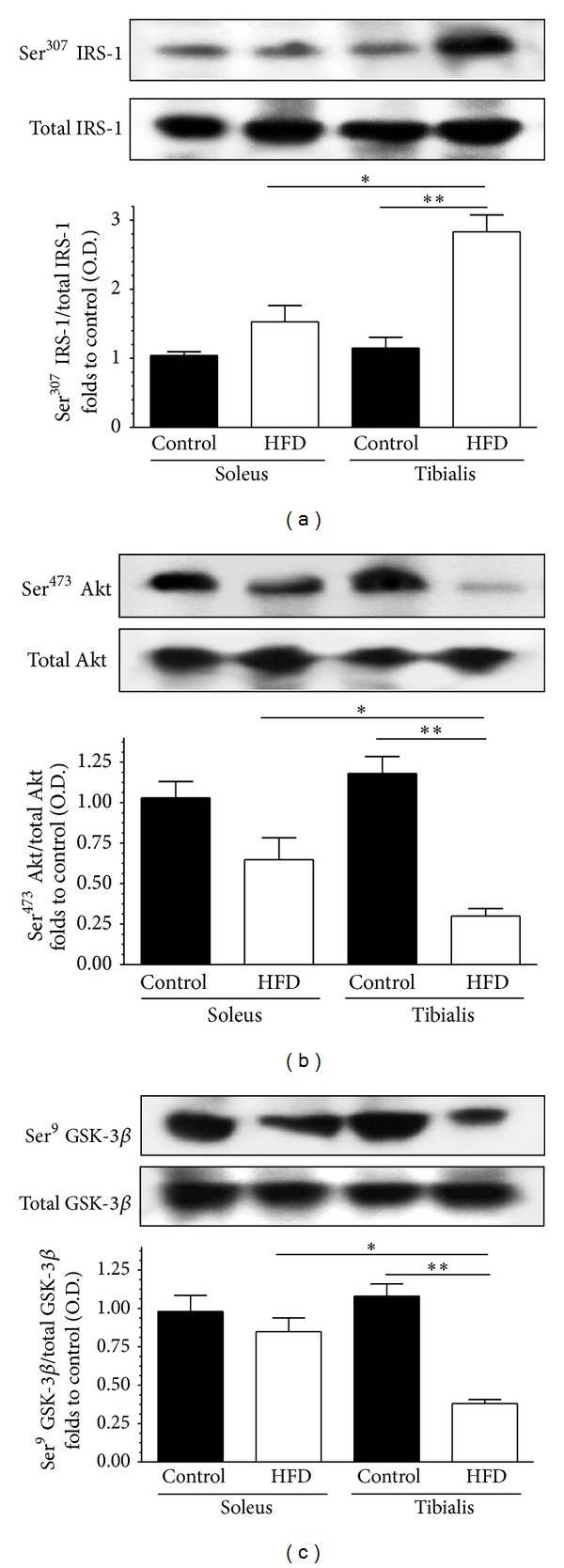
Effect of high-fat diet (HFD) on insulin signal transduction in the soleus and tibialis anterior muscles of control mice and HFD mice. Total IRS-1 protein expression and Ser^307^ phosphorylation (panel (a)), total Akt protein expression and Ser^473^ phosphorylation (panel (b)), and total GSK-3*β* protein expression and Ser^9^ phosphorylation (panel (c)) were analyzed by Western blot on muscle homogenates. Densitometric analysis of the bands is expressed as relative optical density (O.D.), corrected for the corresponding tubulin contents and normalized using the related control band. The data are means ± SD of 10 animals. *P < 0.05; **P < 0.01.

Changes in the activity of the insulin signal transduction pathway were evaluated by immunoblotting experiments on homogenates from both soleus and tibialis anterior muscles (Figure 6). The HFD did not alter the protein expression of the IRS-1, Akt, or GSK-3*β* in both muscles. However, when compared with the tibialis anterior of control mice, HFD mice showed a significant increase in Ser^307^ phosphorylation of IRS-1 in parallel with reduced Ser^473^ phosphorylation of Akt. Ser^9^ phosphorylation of GSK-3*β*, a downstream target of Akt, was also reduced in the tibialis anterior of HFD mice compared to control animals. In contrast, HFD feeding exerted only mild effects on protein phosphorylation in soleus: none of the HFD-induced changes were statistically significant.

## 4. Discussion

In the present study we showed in mice that HFD feeding for 12 weeks caused body weight increase, dyslipidaemia, hyperinsulinaemia, and hyperglycemia. These effects were associated with significant changes in muscle mass, structure, contractile properties, and exercise capacity. Although a number of studies have previously shown that prolonged exposure to elevated levels of fatty acids leads to insulin resistance and impaired muscle glucose and lipid metabolism [4, 14, 15], here we documented that these changes were muscle-specific as the HFD-induced myosteatosis and insulin resistance were more pronounced in the tibialis anterior muscle compared to the soleus muscle.

### 4.1. Effects of HFD on Muscle Mass, Structure, and Function

Mice exposed to HFD increased body weight, decreased muscle mass, and increased muscle triglyceride content. These changes were associated with the following adaptations in muscle contractility and fatigability: the time to peak tension increased, the specific tetanic force decreased, and the fatigue index was reduced in HFD mice compared to control mice.

As differences in skeletal muscle composition significantly affect muscle function, we chose to study diet-induced adaptations in two muscles characterized by high dependence on either oxidative metabolism, the soleus (which mostly contains slow fibers), or glycolytic metabolism, the tibialis (which mostly contains fast fibers) [6–8]. Although we did not assess the HFD-induced fast-to-slow transition of muscle fibers, changes in muscle composition plausibly occurred, as previously documented [2, 3], thus explaining, at least in part, the adaptations in muscle contractile properties. In fact, the tibialis anterior muscle of HFD mice was slower and weaker than that of control mice and the fatigue index was lower in HFD mice compared to control mice. However, resistance to fatigue (assessed by treadmill tests) was lower in HFD mice compared to control mice possibly because the body composition was significantly impaired by HFD in the former group (that showed increased body mass and decreased muscle mass) compared to the latter group of animals.

Defects in mitochondrial functions have been identified in skeletal muscles of obese and/or type 2 diabetic patients [16–18]. Here we observed that mitochondrial changes (i.e., changes in markers of mitochondrial production and activity) were elicited by dietary manipulation, providing a further indirect evidence that a shift toward a greater contribution of oxidative fibers represents a pathophysiological adaptation to high-fat feeding. Interestingly, this adaptation mainly involves fast muscles such as the tibialis anterior. Although the fast-to-slow muscle fiber type transition has previously been documented (and characterized) in response to several conditions (e.g., increased neuromuscular activity, mechanical loading, and hypothyroidism), the molecular mechanism(s) underlying the changes in muscle composition in response to HFD still need to be clarified.

### 4.2. Effects of HFD on Muscle Lipid Metabolism

Tibialis anterior of control mice presented lower triglyceride content than the soleus. This is in agreement with previous findings demonstrating that oxidative muscles store higher amount of lipids than glycolytic muscles, being lipid oxidation their preferential source of energy [19, 20]. Interestingly, when animals were exposed to dietary manipulation, the triglyceride content of the two muscles was comparable, thus suggesting that the glycolytic muscles are more susceptible to alteration evoked by chronic high-fat exposure. Similarly, the elevation of plasma free fatty acids in response to starvation has been reported to lead to muscle-specific responses in intramyocellular fatty acid metabolism. Specifically, starvation evoked increases in intramyocellular lipid levels in glycolytic muscles, but not in oxidative muscles [21, 22]. Notably, a similar intermuscle variability in starvation-induced dynamics of intramyocellular lipid levels has also been reported in humans [23]. We also documented a greater activation of ACC, suggesting increased triglyceride synthesis, in the tibialis compared to the soleus of HFD mice. Although the mechanisms underlying the intermuscle variability in HFD-induced myosteatosis are presently not defined, these findings confirm that oxidative muscles dispose free fatty acids by oxidation when their plasma availability is elevated. In contrast, glycolytic muscles neutralize free fatty acids by reesterification to triglycerides [24, 25]. However, whether myosteatosis represents either a marker of the mitochondrial adaptation/dysfunction or a result of the impaired glucose and lipid oxidation remains to be established. In the latter case, it may be hypothesized that fast muscle fibers are more prone to the intramyocellular triglyceride accumulation due to their lower mitochondrial oxidative phosphorylation reserve compared to slow muscle fibers.

### 4.3. Effects of HFD on Glycometabolic Balance and Muscle Insulin Sensitivity

The intramyocellular triglyceride accumulation was associated with whole body insulin resistance, as documented (by the glucose tolerance test) in the present investigation as well as in previous studies [3, 25, 26]. A novelty of our study compared to previous findings is the assessment of the profile of insulin sensitivity of individual muscles. Previous studies have shown that impairment in insulin signaling is a major component of skeletal muscle insulin resistance [13, 27, 28]. Here we documented that HFD-induced impairments in activation (i.e., phosphorylation) of IRS-2 and the downstream key insulin signaling kinases, Akt and GSK-3*β* (an Akt substrate), were more relevant in the tibialis anterior muscle compared to the soleus muscle. In skeletal muscle cells, insulin-stimulated glucose transport has been shown to occur through the translocation of GLUT-4 from the intracellular pool to the plasma membrane. Therefore, a decreased insulin signaling should imply a defect in insulin stimulation of the GLUT-4 translocation. Consistently, we observed that GLUT-4 translocation to the membrane was markedly inhibited in the tibialis and slightly reduced in the soleus of HFD mice compared to control mice. Importantly, the existence of an association between intramyocellular triglyceride accumulation and insulin-resistance does not imply causality. As previously reported, muscle triglycerides are unlikely to directly cause insulin resistance primarily because they are located within lipid droplets. Instead, it has been proposed that in skeletal muscle they protect fibers from the deleterious actions of other lipid metabolites such as diacylglycerol, ceramides, and long chain acyl-CoAs whose levels can be increased through diet or other perturbations (e.g., lipid and heparin infusion and exercise) [26, 29]. However, we may speculate that myosteatosis changes the composition of the low-density microsomal membranes and thus impairs the insulin-stimulated translocation of GLUT-4 from these intracellular membranes to the plasma membrane. Overall, our study substantiates the previous findings by Shortreed et al. [3] that skeletal muscles respond selectively to high-fat diet intervention and extends our understanding of skeletal muscle adaptive response to dietary insults in keeping to their specific oxidative capacity. As the kinetics and composition of dietary manipulation may be critical, further investigation are warranted to determine whether different types and/or duration of dietary manipulation may induce disturbances similar to those found in our study.

### 4.4. Conclusions

Besides the (causal or protective) effects of myosteatosis on insulin resistance, we found in mice that the soleus muscle stores more triglycerides and is more insulin sensitive than the tibialis anterior muscle in control animals, while the triglyceride content of the two muscles was comparable in HFD mice. A pathophysiological adaptation to lipotoxicity was probably represented by the observed mitochondrial changes that were possibly paralleled by changes in muscle composition and function. These observations deserve future confirmations also in human studies, as to our knowledge no human investigation has previously been performed to assess whether muscle-specific adaptations occur in response to HFD-induced myosteatosis.

Clarifying the cellular and molecular factors modulating the expression and posttranslational control of key proteins in triglyceride metabolism may unmask novel approaches to safely manipulate muscle triglyceride content and the associated insulin resistance.

## Figures and Tables

**Table 1:** Body mass, muscle mass, and glycometabolic parameters in control mice and high-fat diet (HFD) mice after 12 weeks of dietary manipulation.

Variable	Control mice (*n* = 10)	HFD mice (*n* = 10)	*P* value
Body weight increase (g)	8.5 ± 1.6	17.2 ± 4.6	2∗10^−5^
Tibialis anterior weight (% body weight)	0.55 ± 0.05	0.48 ± 0.06	0.021
Soleus weight (% body weight)	0.067 ± 0.009	0.051 ± 0.007	0.002
Plasma glucose (mg/dL)	73 ± 19	120 ± 21	0.008
Plasma insulin (mg/mL)	85.8 ± 5.3	106.0 ± 12.6	0.016
Plasma total cholesterol (mg/dL)	77.2 ± 5.7	129.8 ± 26.3	0.008
Plasma triglycerides (mg/dL)	32.8 ± 7.8	77.0 ± 33.5	0.017
